# Genome-wide identification and characterization of DCL, AGO and RDR gene families in *Saccharum spontaneum*

**DOI:** 10.1038/s41598-020-70061-7

**Published:** 2020-08-06

**Authors:** Dong-Li Cui, Jian-Yu Meng, Xiao-Yan Ren, Jing-Jing Yue, Hua-Ying Fu, Mei-Ting Huang, Qing-Qi Zhang, San-Ji Gao

**Affiliations:** 1 National Engineering Research Center for Sugarcane, Fujian Agriculture and Forestry University, Fuzhou, 350002 Fujian China; 2 College of Agriculture, Fujian Agriculture and Forestry University, Fuzhou, 350002 Fujian China; 3 FAFU and UIUC-SIB Joint Center for Genomics and Biotechnology, Fujian Provincial Key Laboratory of Haixia Applied Plant Systems Biology, Fujian Agriculture and Forestry University, Fuzhou, 350002 Fujian China

**Keywords:** Plant stress responses, Functional genomics

## Abstract

RNA silencing is a conserved mechanism in eukaryotic organisms to regulate gene expression. Argonaute (AGO), Dicer-like (DCL) and RNA-dependent RNA polymerase (RDR) proteins are critical components of RNA silencing, but how these gene families’ functions in sugarcane were largely unknown. Most stress-resistance genes in modern sugarcane cultivars (*Saccharum* spp.) were originated from wild species of *Saccharum*, for example *S. spontaneum*. Here, we used genome-wide analysis and a phylogenetic approach to identify four *DCL*, 21 *AGO* and 11 *RDR* genes in the *S. spontaneum* genome (termed *SsDCL*, *SsAGO* and *SsRDR*, respectively). Several genes, particularly some of the *SsAGOs*, appeared to have undergone tandem or segmental duplications events. RNA-sequencing data revealed that four *SsAGO* genes (*SsAGO18c, SsAGO18b, SsAGO10e* and *SsAGO6b*) and three *SsRDR* genes (*SsRDR2b*, *SsRDR2d* and *SsRDR3*) tended to have preferential expression in stem tissue, while *SsRDR5* was preferentially expressed in leaves. qRT-PCR analysis showed that *SsAGO10c*, *SsDCL2* and *SsRDR6b* expressions were strongly upregulated, whereas that of *SsAGO18b*, *SsRDR1a*, *SsRDR2b/2d* and *SsRDR5* was significantly depressed in *S. spontaneum* plants exposed to PEG-induced dehydration stress or infected with *Xanthomonas albilineans*, causal agent of leaf scald disease of sugarcane, suggesting that these genes play important roles in responses of *S. spontaneum* to biotic and abiotic stresses.

## Introduction

RNA silencing, also known as RNA interference (RNAi), plays an important role in multiple processes in plants, including growth and development, epigenetic modifications and responses to and defenses against abiotic and biotic stresses^1–3^. Several essential steps and core components of RNA silencing pathways are well-characterized. RNA silencing is initially triggered by the formation of double stranded RNAs (dsRNAs) that are subsequently cleaved by the RNase III-type DICER-LIKE proteins (DCL) into small RNA duplexes (sRNAs) of 21–24 nucleotides that include short-interfering RNAs (siRNAs) and microRNAs (miRNAs)^4,5^. Diverse sRNAs are denatured and incorporated into the multi-component RNA-induced silencing complex (RISC) having an Argonaute (AGO) protein at its catalytic core^6,7^. The RISC binds complementary mRNAs guided by single-stranded sRNAs to mediate processes such as translational inhibition, RNA degradation or chromosome modification^8,9^. These sRNAs are amplified from the targeted RNA by cellular host RNA-dependent RNA polymerases (RDRs) to produce additional dsRNAs that will be processed into secondary siRNAs that amplify the silencing signal^10,11^. Notably, RNA silencing-based immunity is also integrated with R gene-mediated immunity in plants for defense against pathogens^12–14^.

The proteins encoded by DCL, AGO, and RDR gene families are core components of the RNA silencing process^5^. DCLs contain a DEAD domain, a helicase conserved C-terminal (Helicase C) domain, a Dicer dimerization domain (Dicer dimer), a PAZ domain (PAZ), a Ribonuclease III domain (Ribonuclease 3), and a double-stranded RNA-binding domain (DSRM)^15,16^. AGOs have four functional domains, i.e., variable MID and N-terminal domains, and conserved PAZ and PIWI domains^7,17^. The PAZ domain can anchor sRNA duplexes with a two-nucleotide 3′ overhang via the specific binding pocket and the PIWI domain that has a similar fold to RNase H and exhibits endonuclease activity, thus playing an important role in target RNA cleavage^18^. RDRs share a special conserved RNA-dependent RNA polymerase (RdRP) catalytic domain^19^. The DCL, AGO, and RDR gene families in plants have species-dependent differences in gene numbers, which range from 20 genes in *Arabidopsis*^20^ to 51 genes in *Brassica* species^21^. Notably, different members of DCL, AGO, and RDR families play different roles in RNA silencing in plants, but they also share partially redundant functions^20^. Currently, there is limited information about DCL, AGO, and RDR gene families in sugarcane.

Sugarcane is an important sugar crop that accounts for 80% of sugar production worldwide and also is one of the most sustainable energy crops that can serve as a biofuel source^22,23^. Modern sugarcane hybrids originated from crosses between *S. officinarum* and one or more other *Saccharum* species and their progenies were progressively backcrossed with different *S. officinarum* clones^24–26^. This process of recurrent introgressive hybridization contributed to commercial hybrids that are highly outcrossed, heterozygous polyploids^26^. Modern sugarcane cultivars offer high sugar content that can primarily be attributed to *S. officinarum*, whereas other traits (e.g., growth vigor, stress resistance, and ratooning) mainly arose from *S. spontaneum*^27–29^. Recently, a genome sequence of the sugarcane wild species clone AP85-441, a haploid *S. spontaneum*, was determined and assembled into 32 pseudo-chromosomes comprising eight homologous groups of four members each ^28^. In addition, a BAC (bacterial artificial chromosome)-based monoploid genome sequence of cultivar R570^27^ and a polyploid genome sequence of cultivar SP80-3280^30^ were also sequenced and assembled. These sugarcane genomic sequences provide valuable reference sequences in the post-genomics era^23^.

Sugarcane often suffers diverse biotic (e.g. pathogenic microorganisms) and abiotic (e.g., drought, cold and high salinity) stresses^22^. Drought stress is one of the most important abiotic stress factors in sugarcane growth and yield worldwide, including in China^31,32^. Additionally, leaf scald caused by *Xanthomonas albilineans* is one of three main sugarcane bacterial diseases, and is responsible for significant loss in cane yield and juice quality^33^. Leaf scald exists in some sugarcane-producing areas in China, where it represents a potential threat to the sugar industry^34,35^. In this study we identified and classified protein members of the DCL, AGO and RDR gene families in the *S. spontaneum* genome, and analyzed the functions of these genes to understand their roles in responses to polyethylene glycol (PEG)-induced dehydration stress and *X. albilineans* infection. Our findings will provide important information for exploration of molecular resistance mechanisms in *S. spontaneum*.

## Methods

### Identification of putative DCL, AGO, and RDR genes in *S. spontaneum*

Hidden Markov Model (HMM) profiles of the characterized and conserved domains of DCL, AGO and RDR families were retrieved from the protein family database (Pfam, https://pfam.xfam.org/)^36^ to search for these gene families in the *S. spontaneum* genome database (https://www.life.illinois.edu/ming/downloads/Spontaneum_genome/)^28^. DCL, AGO, and RDR protein sequences were identified based on the HMM profiles using HMMER software with default parameters^37^ and a cut-off value of 0.01^38^. To ensure the complete identification of the three gene families, we further Blasted DCLs, AGOs and RDRs from maize genome sequences^39^ against the *S. spontaneum* genome database. Conserved domains in all candidate genes were examined using the Pfam and Simple Modular Architecture Research Tool (SMART, https://smart.embl-heidelberg.de/) program. Sequence length, molecular weight and the isoelectric point of DCL, AGO and RDR proteins were predicted using tools at the ExPasy website (https://web.expasy.org/protparam/).

### *In-silico* analysis of gene structure, promoter *cis-*acting elements and protein-protein interactions

Information concerning conserved domains among DCL, AGO and RDR genes in *S. spontaneum*, termed *SsDCL*, *SsAGO* and *SsRDR*, including the domain name and position were obtained by SMART (https://smart.embl-heidelberg.de/). The domain structure and exon–intron organization of the three gene families were analyzed using the online program Gene Structure Display Server (GSDS2.0: https://gsds.cbi.pku.edu.cn)^40^. One kilobase (kb) upstream region from the initial codon of each candidate gene in the three gene families was used to search *cis-*elements by the PlantCARE program (https://bioinformatics.psb.ugent.be/webtools/plantcare/html/). Network analysis of protein–protein interactions (PPIs) among all identified SsDCLs, SsAGOs and SsRDRs was performed using STRING v11.0 (https://string-db.org/) and corresponding maize proteins as reference sequences ^41^. The minimum required interaction score was 0.400, corresponding to medium confidence.

### Chromosomal localization and phylogenetic analysis

The physical locations of *SsDCL*, *SsAGO*, and *SsRDR* genes were determined from the *S. spontaneum* genome database. The chromosomal positions of the three gene families were mapped using Circos software^42^. Gene duplication events were analyzed using the Multiple Collinearity Scan toolkit (MCScanX) with default parameters^43^. Multiple sequence alignments with the respective protein family from *Arabidopsis*^21^, rice^44^, maize^39^ and *S. spontaneum* were performed using the ClustalW program in MEGA 7.0 software^45^. Phylogenetic trees were constructed using a Neighbor-Joining (NJ) method with 1,000 bootstrap replications.

### Plant materials and experimental treatments

Cuttings of *S. spontaneum* clone SES208 were provided by the Center for Genomics and Biotechnology, Fujian Agriculture and Forestry University (Fuzhou, China). Clone SES208 is an octoploid donor used to generate the haploid AP85-441 clone through anther cultures^26^. The cuttings were grown in a plant growth chamber under a 16 h/8 h light/dark period at 30 °C and 70% relative humidity (RH). Four-week-old plants (three leaves fully expanded) of clone SES208 were used in two experimental treatments: drought stress and bacterial stress. Roots of 24 plants were immersed in a 25% PEG-6000 solution for 0, 3, 6, and 12 h and respective top young leaf samples were collected. Leaves from another 24 plants were inoculated with *X. albilineans* strain Xa-FJ1 following the protocol described by Lin et al.^34^ and inoculated leaves were collected at 0, 24, 48, and 72 h post inoculation (hpi). All samples were frozen in liquid nitrogen and stored at − 80 °C until total RNA was extracted.

### Quantitative real-time PCR (qRT-PCR)

Total RNA from leaf tissues was extracted by TRIzol reagent (Invitrogen/Life Technologies, Carlsbad, CA, USA) according to the manufacturer’s instructions. RNA quality was analyzed by electrophoresis on a 1% agarose gel and RNA amounts were quantified using a Synergy™ H1 hybrid multimode reader (BioTek, Winooski, VT, USA). All RNA samples were diluted to a working concentration of 1.0 µg/µL with RNase-free H_2_O for further analysis. Total RNA (1.0 µg) was used in reverse transcription (RT) reactions to generate the first strand cDNA by HiScript II RT (Hongfeng Science and Technology, Nanjing, China) with random primers following the manufacturer’s directions. Because of the high homology in some gene pairs, we could not design primers with high specificity for every gene members, thus nine, three, and six candidate genes from *SsAGOs*, *SsDCLs*, and *SsRDRs*, respectively, were chosen to represent the different sub-families for quantitative real time PCR (qRT-PCR) transcriptional expression analyses. These genes primer pairs were designed with the GenScript Real-time PCR (TaqMan) Primer Design tool (https://www.genscript.com/tools/real-time-pcr-taqman-primer-design-tool) (Table S1). The qRT-PCR reactions were performed using 94 °C for 30 s followed by 40 cycles of 95 °C for 10 s and 60 °C for 30 s. The housekeeping gene, glyceraldehyde 3-phoshate dehydrogenase (*GAPDH*), was used as an internal control for normalization. The qRT-PCR results were analyzed by the 2^-∆∆Ct^ quantitative method to determine differences in gene expression^44^. Three biological and three technical replicates were carried out for each sample.

### Data analysis

Relative expression levels determined from qRT-PCR data at different time points for each cultivar were analyzed using one-way ANOVA. Multiple comparisons of the means were conducted by the SNK (Student–Newman–Keuls) Test. All statistical analyses were carried using SAS version 8.1 (SAS Institute, Cary, NC, USA).

## Results

### Identification and structural analysis of SsAGO, SsDCL, and SsRDR genes

To identify the AGO, DCL, and RDR gene families in *S. spontaneum*, we gathered the previously characterized and conserved domains of the three gene families and used HMMER software to search for corresponding domains in the *S. spontaneum* genome database. In addition, members of the three gene families from maize genome sequences were blasted against the *S. spontaneum* genome database. After evaluation of the structural integrity of conserved domains and elimination of redundant sequences, four genes encoding DCL proteins (SsDCLs), 21 genes encoding AGO proteins (SsAGOs) and 11 genes encoding RDR proteins (SsRDRs) were identified in *S. spontaneum* (Table S2). The detailed characteristics of all genes identified in this study, including chromosomal location and protein properties (e.g., open reading frame (ORF) length, protein length (amino acid, aa), molecular weight (MW), and isoelectric point (IP)) are listed in Table 1.

**Table 1:** Structural characteristics and physio-chemical properties of dicer-like (DCL), argonaute (AGO) and RNA dependent RNA polymerase (RDR) genes from *S. spontaneum*.

Gene name	Gene ID	Location			Protein			
		Chromosome	Start	End	CDS (bp)	Length (aa)	Mw (Da)	PI
**Dicer-like (DCL)**								
SsDCL1a	Sspon.01G0001230-3D	Chr1D	3,692,925	3,701,074	4,377	1,458	163,204.71	6.05
SsDCL1b	Sspon.01G0001230-1A	Chr1A	3,976,165	3,983,525	4,770	1,590	178,065.62	6.05
SsDCL2	Sspon.01G0022370-1A	Chr1A	81,960,057	81,976,273	3,699	1,233	139,512.97	7.06
SsDCL3	Sspon.01G0019860-4D	Chr1D	71,661,374	71,672,007	4,752	1584	179,009.05	6.24
**Argonaute (AGO)**								
SsAGO2a	Sspon.05G0030950-2D	Chr5D	11,880,179	11,884,678	3,066	1,021	111,963.65	8.73
SsAGO2b	Sspon.05G0037340-1D	Chr5D	11,924,233	11,928,734	2,763	921	100,646.66	7.81
SsAGO3a	Sspon.05G0030960-1C	Chr5C	2,396,093	2,401,132	3,048	1,015	110,049.24	9.30
SsAGO3b	Sspon.05G0030950-1C	Chr5C	2,386,282	2,391,173	2,904	967	104,620.95	9.27
SsAGO3c	Sspon.05G0030950-1P	Chr5D	12,513,102	12,517,597	3,036	1,011	109,860.1	9.35
SsAGO4	Sspon.02G0016200-2D	Chr3D	39,536,146	39,547,268	2,838	946	105,523.71	8.60
SsAGO5a	Sspon.01G0028360-2B	Chr1B	96,848,274	96,854,895	3,165	1,054	115,793.38	9.42
SsAGO5b	Sspon.01G0014460-3D	Chr1D	87,130,069	87,135,808	2,742	913	102,643.41	9.28
SsAGO5c	Sspon.01G0014460-1A	Chr1A	41,213,733	41,219,461	2,418	806	90,379.42	9.55
SsAGO5d	Sspon.01G0014460-2B	Chr1B	96,914,953	96,920,845	2,784	927	103,595.76	9.55
SsAGO6a	Sspon.07G0020900-1A	Chr7A	78,332,899	78,336,592	1,902	634	70,983.26	9.46
SsAGO6b	Sspon.07G0020900-2D	Chr7D	60,984,388	60,994,488	2,070	689	77,218.74	9.34
SsAGO10a	Sspon.08G0006580-1P	Chr8D	17,872,226	17,876,344	2,271	757	83,975.06	9.22
SsAGO10b	Sspon.08G0006580-1A	Chr8A	20,403,262	20,409,634	2,829	942	105,347.29	9.47
SsAGO10c	Sspon.08G0006580-2B	Chr8B	18,085,409	18,091,645	2,394	797	88,300.59	9.67
SsAGO10d	Sspon.08G0006580-4D	Chr8D	17,765,548	17,771,337	1,884	628	71,264.01	9.40
SsAGO10e	Sspon.08G0006580-3C	Chr8C	9,379,029	9,385,180	2,916	971	108,586.36	9.43
SsAGO18a	Sspon.02G0007830-1A	Chr2A	22,748,211	22,753,686	3,099	1,032	113,076.36	9.47
SsAGO18b	Sspon.01G0024300-1A	Chr1A	87,229,183	87,234,462	2,382	793	88,734.95	9.29
SsAGO18c	Sspon.01G0024300-3D	Chr1D	84,919,502	84,924,770	2,364	787	88,118.01	9.00
SsAGO18d	Sspon.02G0007830-2D	Chr2D	16,391,380	16,396,270	2,754	918	100,666.07	9.44
**RNA dependent RNA Polymerase (RDR)**								
SsRDR1a	Sspon.04G0003980-2D	Chr4D	12,916,500	12,921,015	3,324	1,107	126,692.84	7.73
SsRDR1b	Sspon.04G0003980-1A	Chr4A	12,309,373	12,314,472	3,348	1,115	127,601.91	7.53
SsRDR2a	Sspon.05G0009620-1A	Chr5A	27,714,217	27,719,638	3,414	1,137	126,512.75	6.67
SsRDR2b	Sspon.05G0009620-2B	Chr5B	22,682,674	22,688,038	3,315	1,104	122,789.35	6.50
SsRDR2c	Sspon.05G0009620-3C	Chr5C	19,106,947	19,112,258	3,414	1,137	126,557.88	6.39
SsRDR2d	Sspon.05G0009620-4D	Chr5D	30,160,562	30,165,865	2,967	989	110,364.29	7.29
SsRDR3	Sspon.05G0014430-3C	Chr5C	48,023,413	48,044,408	2076	691	79,290.78	7.29
SsRDR4	Sspon.05G0014430-4D	Chr5D	52,146,251	52,168,613	2049	683	77,901.79	6.25
SsRDR5	Sspon.03G0023160-2C	Chr3C	89,299,759	89,307,281	2,514	838	96,153.93	8.70
SsRDR6a	Sspon.08G0019290-1B	Chr8B	11,200,927	11,205,278	3,240	1,079	120,677.59	7.10
SsRDR6b	Sspon.08G0019290-2D	Chr8D	11,219,834	11,222,839	3,006	1,001	111,860.59	8.24

*CDS* coding sequence, *MW* molecular weight, *IP* isoelectric point.

**Figure 1:**
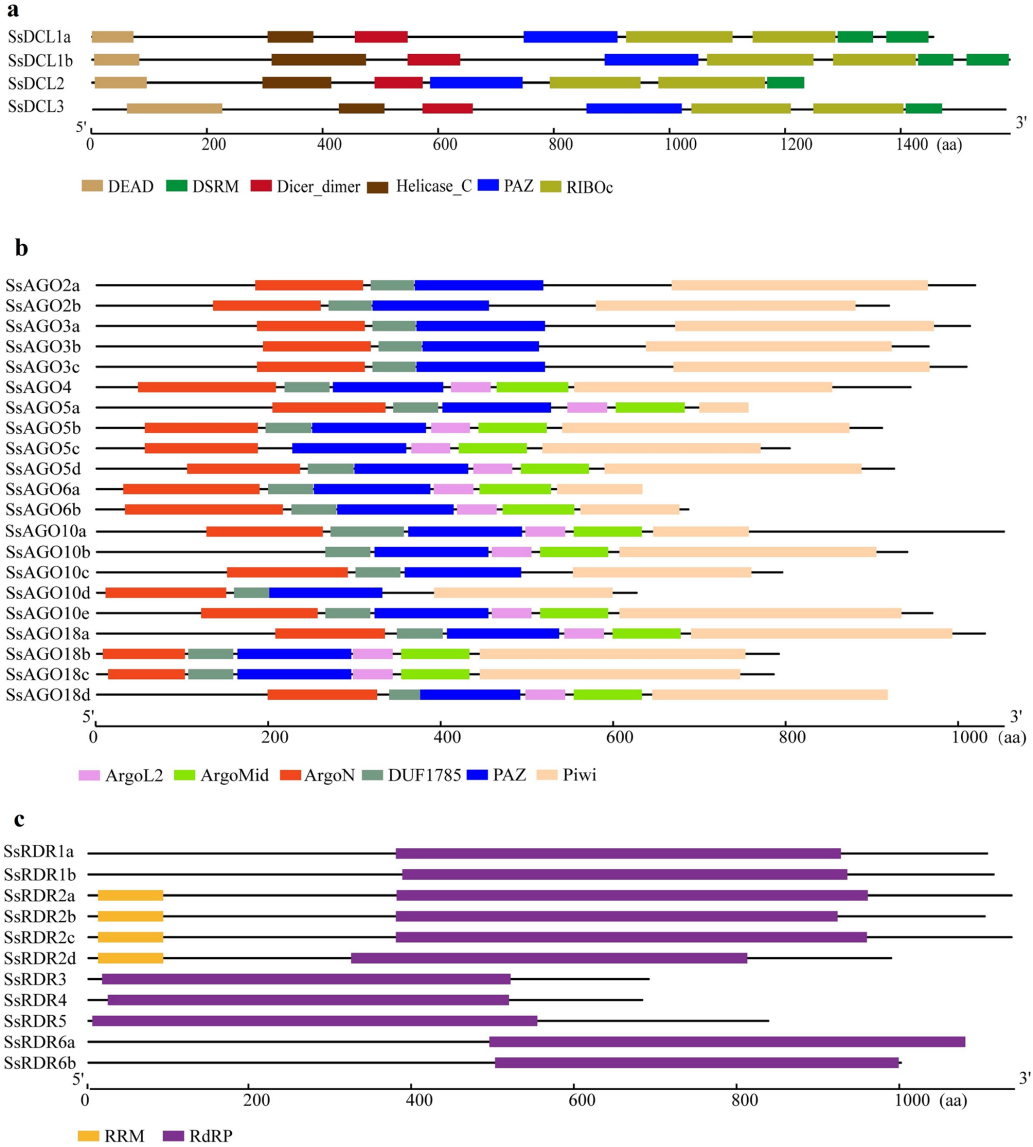
Structural domains of dicer-like protein (SsDCL) (**a**), argonaute protein (SsAGO) (**b**), and RNA-dependent RNA polymerase (SsRDR) (**c**) from *S. spontaneum*. Domains are indicated by colored boxes. The scale bars at the bottom represent the length of proteins in aa (amino acid).

The proteins encoded by the four identified SsDCLs ranged from 1,233 aa (*SsDCL2*, Sspon.01G0022370-1A) to 1,590 aa (*SsDCL1b*, Sspon.01G0001230-1A) and contained a Dicer_dimer domain, a PAZ domain, a Helicase_C domain, a DEAD domain, a RIBOc domain and one or two DSRM domains (one for SsDCL2/3, and two for SsDCL1a/b) (Fig. 1A). The 21 identified SsAGOs ranged from 628 aa (*SsAGO10d*, Sspon.08G0006580-4D) to 1,054 aa (*SsAGO5a*, Sspon.01G0028360-2B), and shared common domains including a DUF1785 domain, PAZ domain, PIWI domain, and ArgoN domain. ArgoL2 and ArgoMid domains that were present in most of the SsAGOs identified (14/21), but were absent in SsAGO2a/b, SsAGO3a/b/c, and SsAGO10c/d (Fig. 1B). SsRDRs varied from 683 aa for *SsRDR4* (Sspon.05G0014430-4D) to 1,137 aa for *SsRDR2c* (Sspon.05G0009620-3C), which contained the common sequence motif for DNA-dependent RNA polymerases (RdRP) whereas SsRDR2a/b/c/d had another common sequence motif, the RRM domain (Fig. 1C). Comparative structural analysis for exons-introns in the three gene families revealed that the number of introns ranged from two in *SsAGO2b/3a* to 22 in *SsAGO4*, from 15 in *SsDCL1a* to 24 in *SsDCL3*, and from one in *SsRDR6b* to 17 in *SsRDR3* (Figure S1).

Furthermore, pairwise sequence alignment and identity analysis revealed that the conversed motifs among these gene members in each DCL, AGO and RDR families were high discrepancy (Figure S2 and Table S3). The identifies of amino acid sequences in DCL, AGO and RDR gene families were 29.7–85.8%, 12.3–99.3% and 11.3–100%, respectively (Table S3). Among gene pairs of alleles, these sequences shared higher identifies with each other. For example, the gene pairs of alleles in SsDCLs, the amino acid identity was 85.8% between *SsDCL1a* and *SsDCL1b*; the gene pairs of alleles in AGOs, the amino acid identities were 91.5% (*SsAGO2a* and *SsAGO2b*), 83.6–91.8% (*SsAGO3a*, *SsAGO3b* and *SsAGO3c*), 60.5–89.5% (*SsAGO5a, SsAGO5b*, *SsAGO5c*, and *SsAGO5d*), 93.2% (*SsAGO6a* and *SsAGO6b*), 43.5–97.3% (*SsAGO10a*, *SsAGO10b*, *SsAGO10c*, *SsAGO10d* and *SsAGO10e*), and 53.0–95.2% (*SsAGO18a*, *SsAGO18b*, *SsAGO18c* and *SsAGO18d*). The gene pairs of alleles in RDRs, the amino acid identities were 100% (*SsRDR1a* and *SsRDR1b*), 77.4–99.6% (*SsRDR2a*, *SsRDR2b*, *SsRDR2c* and *SsRDR2d*) and 83.7% (*SsRDR6a* and *SsRDR6b*).

### Prediction of *cis-*acting elements in the putative gene promoters of SsAGO, SsDCL and SsRDR

**Figure 2:**
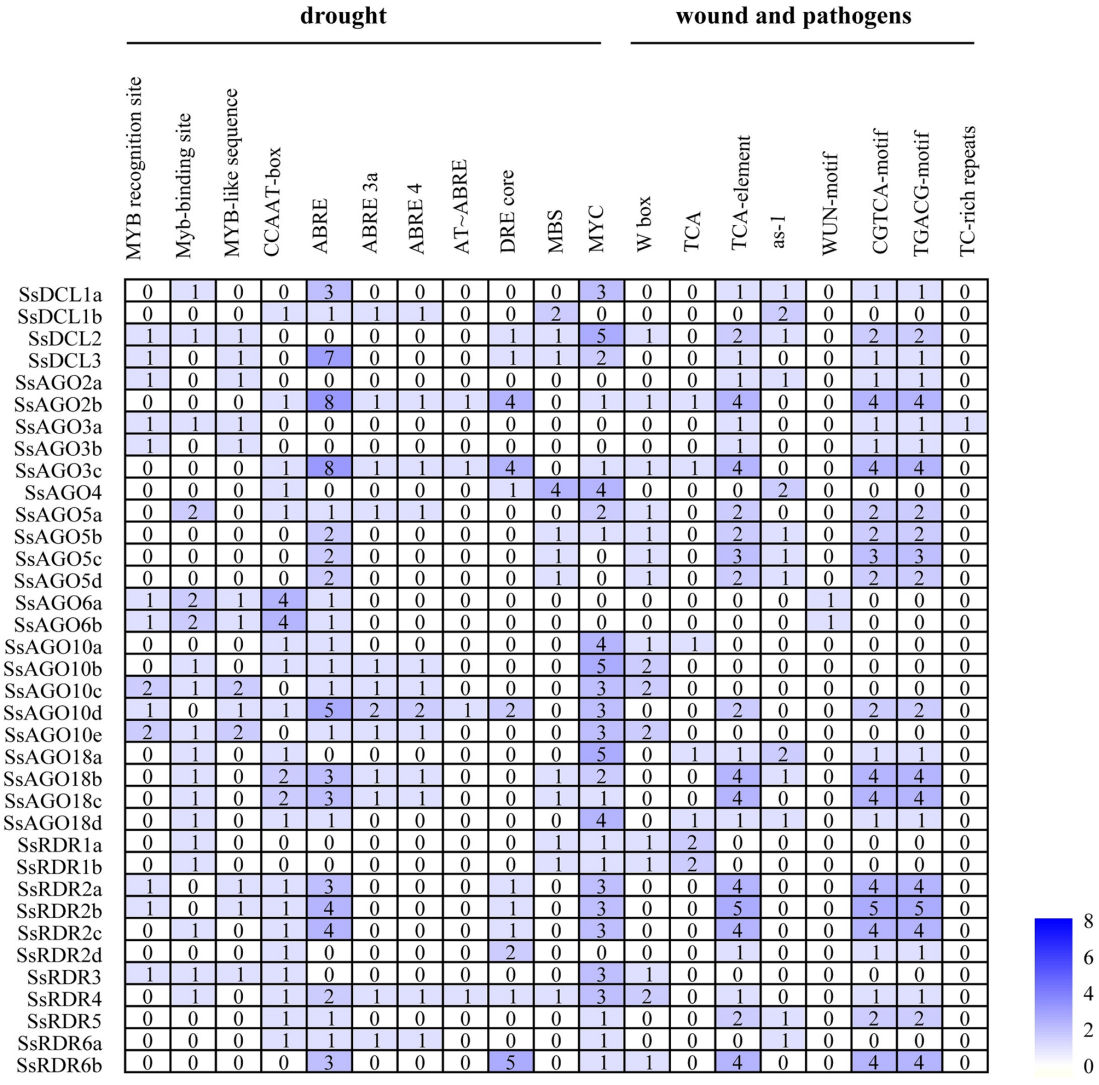
Analysis of putative *cis-*acting elements related to response to drought or wound and pathogen stresses in *S. spontaneum* promoter sequences (1 kb) of *SsDCL*, *SsAGO*, and *SsRDR* genes. Numbers of elements present are indicated with darker blue shading representing higher numbers.

Sequences (1 kb) of upstream of the translation initiation codon for the *SsAGO*, *SsDCL*, and *SsRDR* genes were examined for the presence of *cis-*acting elements using the PLANTCARE online database. In addition to *cis-*acting elements that are characteristic of eukaryotic promoters, various *cis-*acting elements including those associated with plant growth, development, and stress responses were found among SsAGO, SsDCL, and SsRDR family members. The upstream sequences of all *SsAGO*, *SsDCL*, and *SsRDR* genes had the *cis-*acting element of eukaryotic promoters (CAAT-box) and, except for *SsAGO2b/10d* and *SsRDR2a/2b/6b*, had a TATA-box that is another *cis-*acting element of eukaryotic promoters (Table S4). Numerous *cis-*acting elements associated with drought/dehydration response including DRE core motifs, MYB recognition or binding sites, MBS, MYC, and ABRE elements were present (Fig. 2). Genes including *SsDCL3*, *SsAGO2b*, *SsAGO3c*, *SsAGO10c/d/e*, *SsAGO18b*, *SsRDR2b*, and *SsRDR4* had more than ten *cis-*elements related to dehydration response. More than three classes of MYC elements were found in *SsDCL2*, *SsAGO3a/b/c*, *SsAGO18a*, *SsRDR1a/b*, *SsRDR2d*, and *SRDR3*, but no MYB elements were predicted in *SsAGO5b*, *SsAGO5d*, and *SsRDR6b*.

Multiple *cis-*acting elements involved in plant wound and pathogen response were also predicted in these promoters. More than one *cis-*acting element (W box, TGACG-motif or TCA-element) related to salicylic acid (SA) response were present in all gene family members, except for *SsAGO6a/b*. Two or more *cis-*acting element sites (CGTCA motif or TGACG motif) related to Methyl Jasmonate (MeJA) regulation presented in 24 promoters among the three gene families. Notably, the *cis-*element W box, which is related to responses to plant pathogen invasion, was present in *SsDCL2*, *SsAGO2b*, *SsAGO3c*, *SsAGO5a/b/c/d*, *SsAGO10a/b/c/e*, *SsRDR1a/b*, *SsRDR3*, *SsRDR4*, and *SsRDR6b*. The TC-rich repeat associated with plant defense against pathogen infection was present in *SsAGO3a*. Meanwhile, the WUN-motif related to mechanical damage was predicted only in the *SsAGO6a/b* promoter.

### Phylogenetic analysis and chromosomal localization

**Figure 3:**
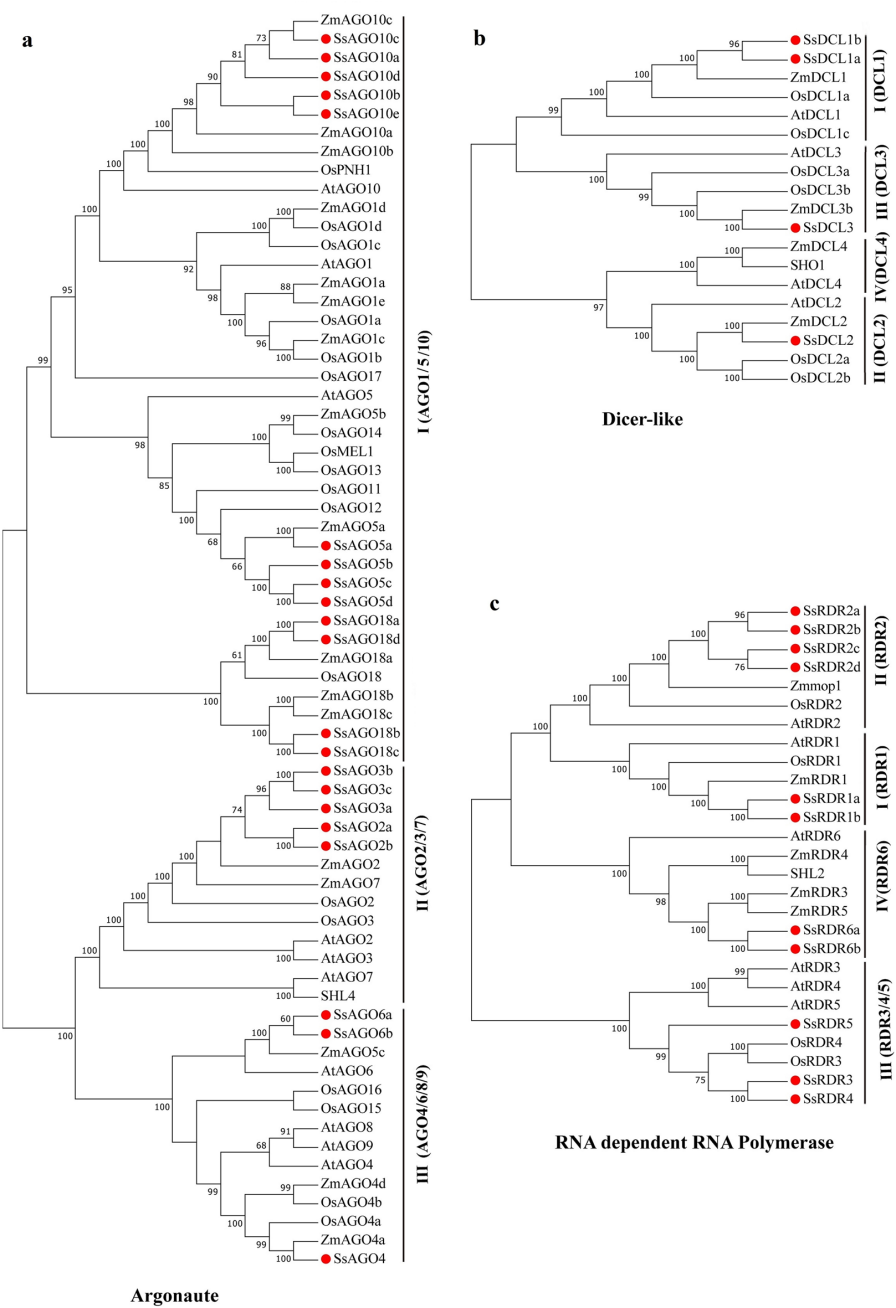
Phylogenetic analysis of *S. spontaneum SsAGO***(a)**, *SsDCL***(b)**, and *SsRDR***(c)** genes. Neighbor-Joining (NJ) trees were constructed using MEGA 7 software based on the protein sequences for each family member. Bootstrap support values from 1,000 replications are indicated above the branches. *S. spontaneum* genes are indicated by a red circle.

To demonstrate phylogenetic relationships among identified genes encoding AGO, DCL, and RDR proteins, Neighbor-joining based phylogenetic trees were constructed using MEGA7 software based on the proteins of the three families from *Arabidopsis*, rice, maize, and *S. spontaneum* (Fig. 3, Table S2). All 19 *DCL* genes analyzed were clustered into four Clades, termed Clade I–IV (DCL1-4). Among the four *SsDCL* genes from *S. spontaneum*, *SsDCL1a/1b* genes were in Clade I, whereas *SsDCL2* and *SsDCL3* were grouped in Clade II and III, respectively. Unexpectedly, no *SsDCL* gene was found in Clade IV. All 59 *AGO* genes analyzed were clustered into three major Clades, including Clade I (AGO1/5/10), Clade II (AGO2/3/7), Clade III (AGO4/6/8/9). It is noteworthy that an AGO18 group from rice, maize and *S. spontaneum* falls into the Clade I. All 27 *RDR* genes were separated into four Clades, namely Clade I (RDR1), Clade II (RDRII), Clade III (RDR3/4/5), and Clade IV (RDR6). The 11 *SsRDR* genes from *S. spontaneum* were present in each of the four Clades.

**Figure 4:**
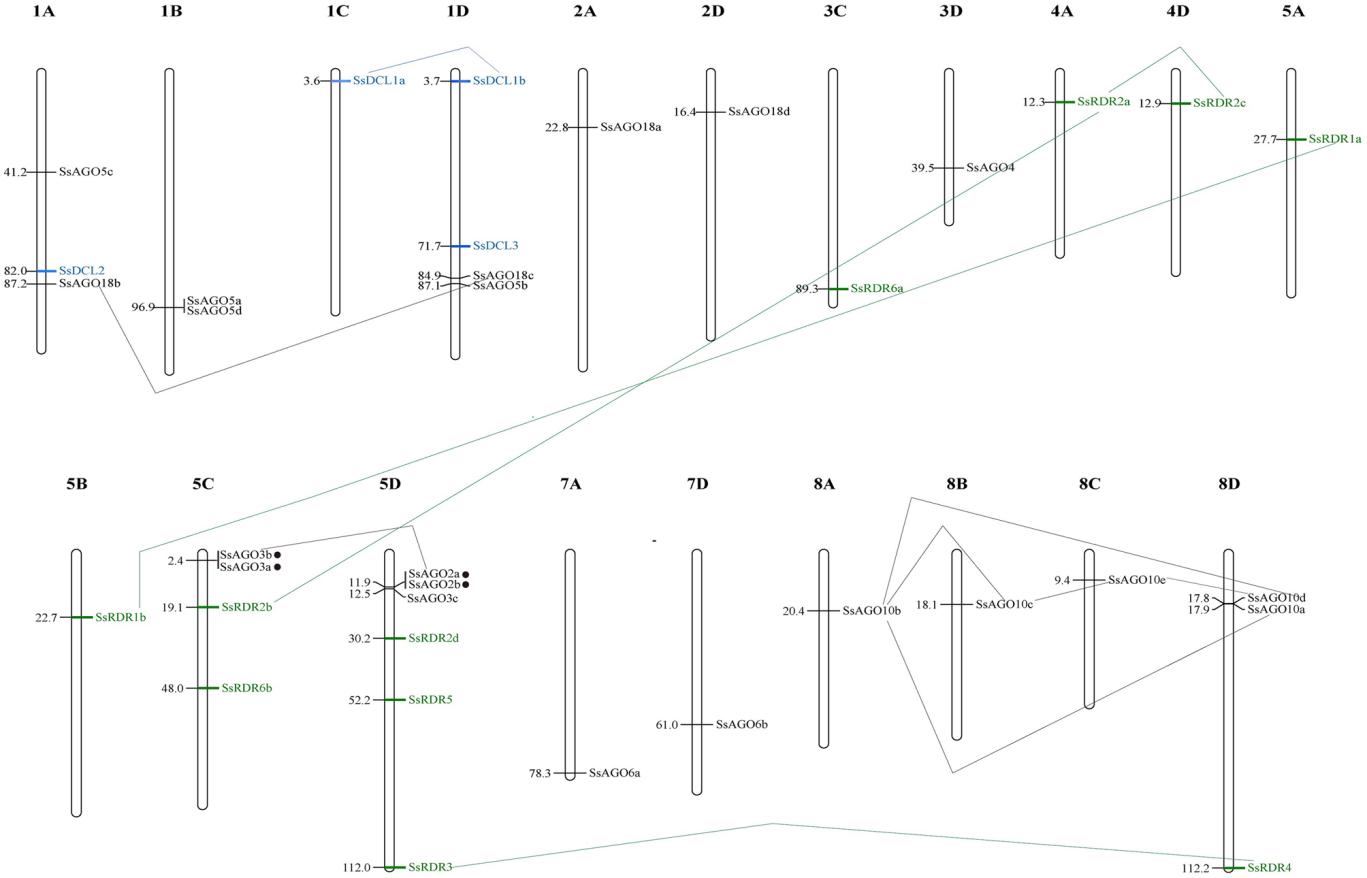
Chromosome localization of *SsDCL* (in blue), *SsAGO* (in black), and *SsRDR* (in green) genes. The chromosome number is shown at the top of each bar. Horizontal bars represent the gene locations on each chromosome with positions in Mb (megabases) shown. Genes having tandem duplications are indicated by solid circles, whereas segmental duplication genes are joined by blue (SsDCLs), black (SsAGOs), and green lines (SsRDRs).

All 36 identified *S. spontaneum* genes encoding DCLs, AGOs, and RDRs were precisely located on 19 of 32 *S. spontaneum* chromosomes (comprising 8 homologous groups of 4 members each): six genes on chromosome 5D, four genes on chromosome 1A, 1D and 5C, three genes on chromosome 8D, two genes on chromosome 1B, and one gene on the other remaining chromosomes (1C, 2A, 2D, 3C, 4A, 4D, 5A, 5B, 7A, 7D, 8A, 8B, and 8C (Fig. 4). Of the three gene families, *SsAGO* genes were widely distributed over thirteen chromosomes, followed by *SsRDR* genes that were distributed over eight chromosomes (3C, 4A, 4D, 5A, 5B, 5C, 5D, 8B, and 8D), and *SsDCL* genes over three chromosomes (1A, 1C, and 1D). Meanwhile, duplication events that occurred over the course of *S. spontaneum* genome evolution were revealed by analysis with the BLASTp tool and MCScanX software. Two pairs of tandem duplicated *SsAGO* genes (*SsAGO2a* vs *SsAGO2b*; *SsAGO3a* vs *SsAGO3b*), which localized to chromosomes 5C and 5D, respectively, shared 91.5% and 83.6% identity with each other at an amino acid level. No tandem duplicated genes were seen for *SsDCLs* and *SsRDRs*. Twelve pairs of segmental duplicated genes were also found among the three families (one pair in *SsDCLs*, seven pairs in *SsAGOs*, and four pairs in *SsRDRs*), indicating that some of these genes could have been generated by gene duplication and that segmental duplication events play a major role in *S. spontaneum* genome evolution.

### Protein–protein interaction networks for SsAGOs, SsDCLs, and SsRDRs

**Figure 5:**
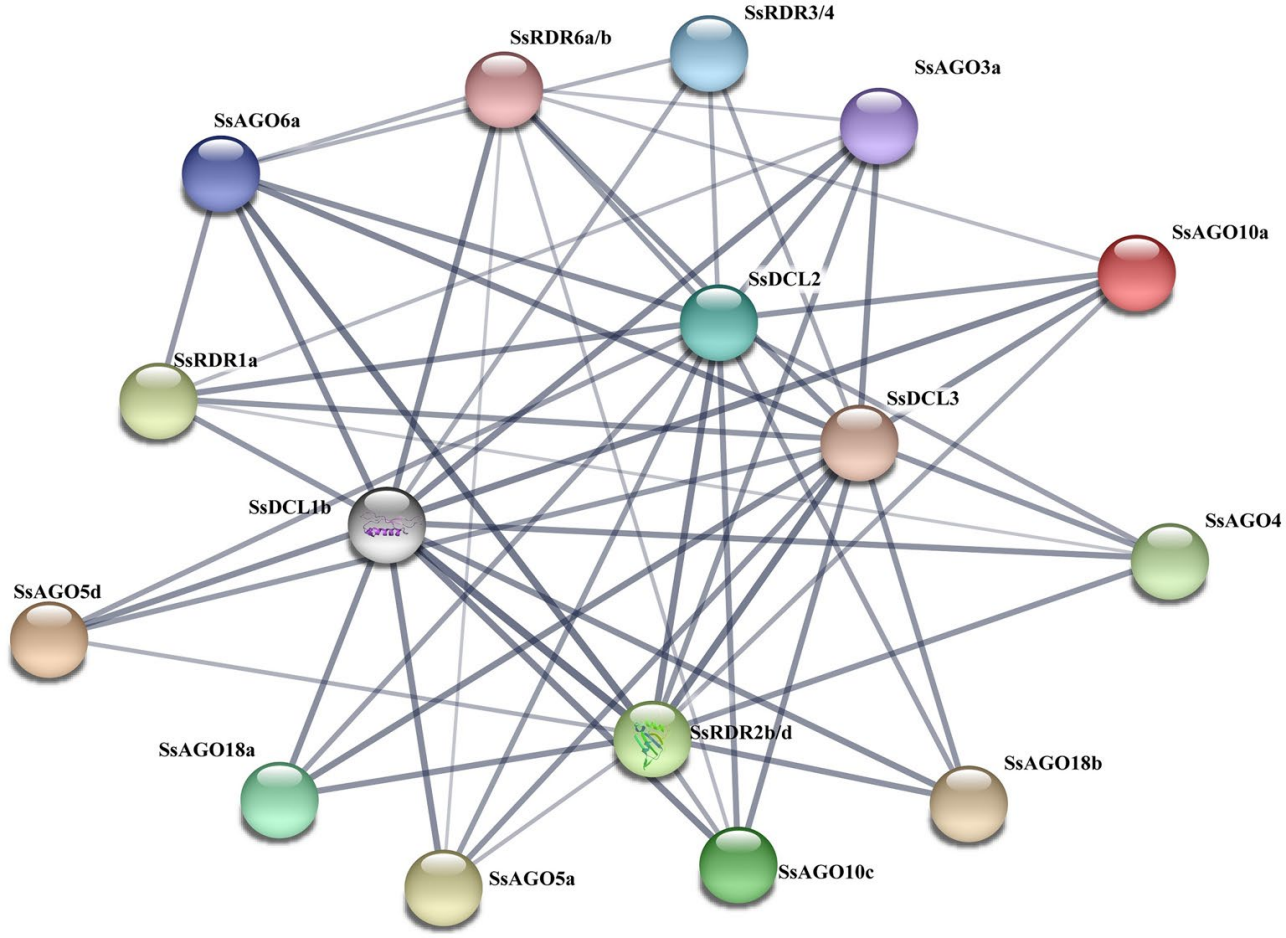
Schematic representation of protein–protein interaction (PPI) networks between SsDCLs, SsAGOs, and SsRDRs from *S. spontaneum*. Nodes having different colors indicate different proteins. Gray lines connect proteins within the PPI networks with darker colors and thicker lines indicating higher core PPI values.

To investigate protein–protein interactions (PPIs) among the 36 proteins (4 SsDCLs, 21 SsAGOs, and 11 SsRDRs) identified for *S. spontaneum*, a PPI network was predicted *in-silico* with the STRING database using maize sequences as queries. PPI network analysis showed that 20 of 36 identified proteins interacted with each other, including SsDCL1b, SsDCL2, SsDCL3, SsAGO3a, SsAGO4, SsAGO5a/d, SsAGO6a/b, SsAGO10a/c, SsAGO18a/b, SsRDR1a, SsRDR2b/d, SsRDR3, SsRDR/4, and SsRDR6a/b (Fig. 5). Some of these proteins (SsDCL1b-SsAGO6a-SsRDR2b/d, SsDCL1b-SsAGO18a-SsRDR2b/d, SsDCL1b-SsAGO6a-SsRDR6a/b, SsDCL1b-SsAGO10a-SsRDR6a/b, SsDCL2-SsAGO3a-SsRDR2b/d, SsDCL3-SsAGO4-SsRDR2b/d, SsDCL3-SsAGO6a-SsRDR2b/d, and SsDCL3-SsAGO18b-SsRDR2b/d) interacted strongly. These results indicated that various combinations of three core components of SsDCLs, SsAGOs, and SsRDRs may participate in different RNA silencing pathways in *S. spontaneum*.

### Expression pattern of SsAGOs, SsDCLs and SsRDRs in leaf and stem tissues

**Figure 6:**
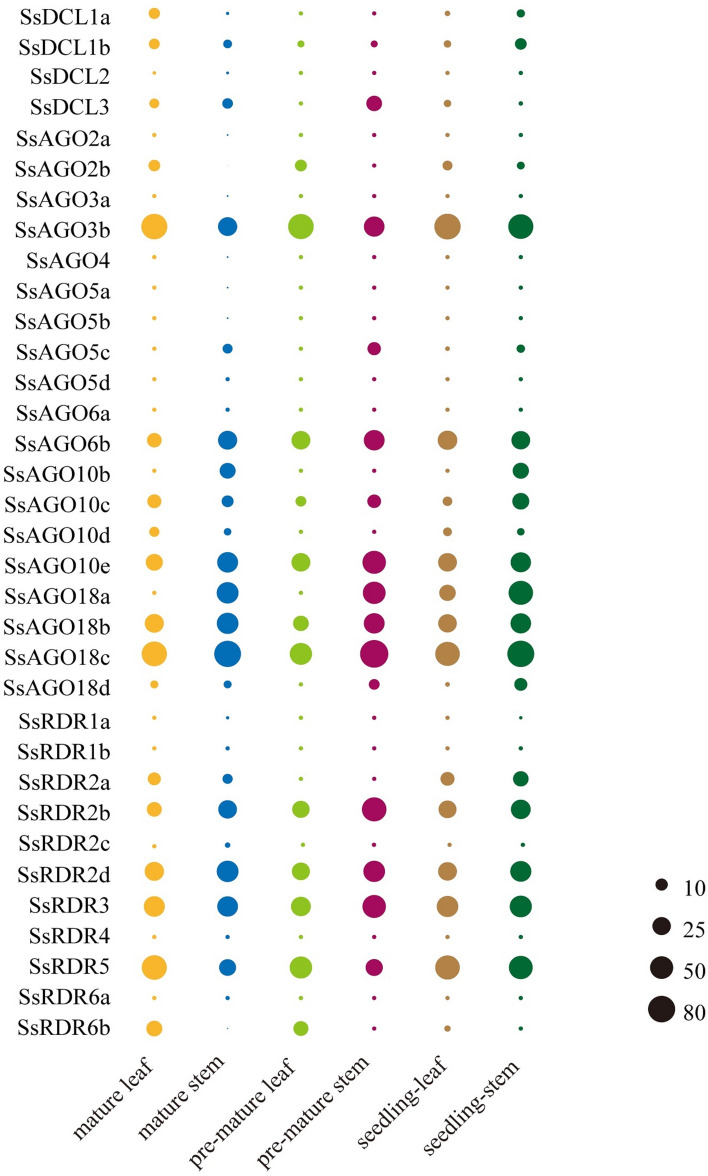
Heat map showing spatiotemporal expression patterns of genes encoding *SsDCL*, *SsAGO*, and *SsRDR* in various *S. spontaneum* tissues including mature leaf, mature stem, pre-mature leaf, pre-mature stem, seedling leaf, and seedling stem. The size of the circles represents normalized expression level wherein larger circles correspond to higher expression levels.

To determine the temporal and spatial expression patterns of genes encoding AGO, DCL, and RDR in *S. spontaneum*, transcriptional expression analysis was performed in leaf and stem samples from seedlings as well as from pre-mature and mature plants using the RNA-sequencing (RNA-seq) database (https://www.life.illinois.edu/ming/downloads/Spontaneum_genome/) (Fig. 6). Overall, no obvious temporal expression pattern was observed among the 36 identified genes, but expression patterns that tended to be specific to some tissues were observed. The four genes encoding SsDCLs exhibited no or very weak expression levels in either leaf or stem tissue. For the *SsAGO* genes, five genes (*SsAGO3b*, *SsAGO18c*, *SsAGO18b*, *SsAGO10e*, and *SsAGO6b*) had high expression levels in both leaf and stem at all growth stages whereas, except for the *SsAGO3b*, showed higher expression levels in stem tissues relative to leaf tissues. The *SsAGO18a* gene was also preferentially highly expressed in stem tissues, but not in leaf tissues, particularly mature or pre-mature leaves. The *SsAGO10c* gene had moderate expression levels, but the others were absent or had very weak expression levels in leaves and stems. Of the *SsRDR* genes, four (*SsRDR2b*, *SsRDR2d*, *SsRDR3*, and *SsRDR5*) exhibited higher expression levels in leaves and stems, whereas another five (*SsRDR1a/b*, *SsRDR2c*, *SsRDR4*, and *SsRDR6a*) had no detectable expression in either tissue type. Notably, of the four high-expression genes, *SsRDR5* had higher expression levels in leaves compared to stems. Conversely, *SsRDR2b*, *SsRDR2d*, and *SsRDR3* genes had higher expression in stems relative to leaves.

### SsAGO, SsDCL, and SsRDR expression patterns induced by polyethylene glycol (PEG)-induced dehydration stress

**Figure 7:**
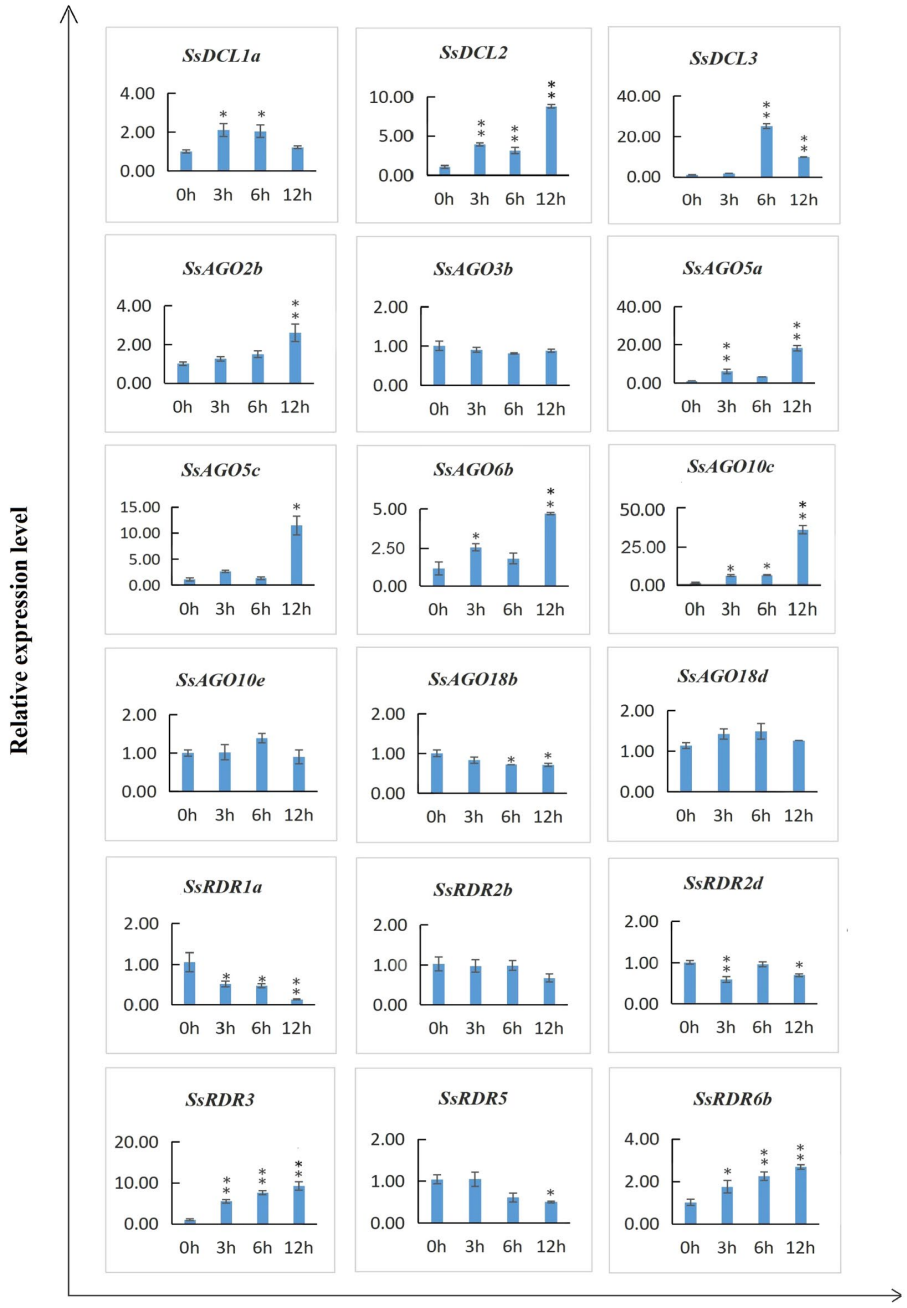
Quantitative real time PCR (qRT-PCR) analysis of relative transcript expression of 18 candidate genes encoding *SsDCL*, *SsAGO*, and *SsRDR* in *S. spontaneum* clone SES208 exposed to dehydration treatment (PEG-6000) for 0, 3, 6, and 12 h. The x-axis indicates the time points of PEG-6000 exposure, whereas the y-axis indicates the relative expression level. The top young leaves were sampled and used for qRT-PCR assay. Relative transcript expression values are presented as means ± standard errors based on three biological replicates with three technical replicates. Significant differential expression is indicated by an asterisk (*, *p* < 0.05; **, *p* < 0.01).

To measure SsAGO, SsDCL**,** and SsRDR expression patterns in *S. spontaneum* under dehydration stress, the transcript expression for 18 candidate genes (9, 3 and 6 *SsAGOs*, *SsDCLs*, and *SsRDRs*, respectively) were analyzed by qRT-PCR upon the young plants of *S. spontaneum* clone SES208 subjected to PEG6000 treatment for 0–12 h(Fig. 7). The expression levels of five *SsAGO* genes (*SsAGO2b, SsAGO5a*, *SsAGO5c*, *SsAGO6b*, and *SsAGO10c*) in top young leaves were significantly upregulated by > 2.5-fold, while *SsAGO5a*, *SsAGO5c*, and *SsAGO10c* had particularly large increases of 18-, 11- and 35-fold, respectively, after 12 h of PEG treatment. Meanwhile, expression of *SsAGO18b* was significantly downregulated after PEG treatment for 6–12 h. In the *SsDCL* family, *SsDCL1a* expression was significantly upregulated by PEG treatment for 3–6 h with an increase of twofold, whereas transcript levels of *SsDCL2* and *SsDCL3* were dramatically increased by 3–ninefold at 3–12 h and 10–25-fold at 6–12 h, respectively, post PEG treatment. In the RDR family, *SsRDR3* and *SsRDR6b* expression level were highly upregulated with increases of 6–ninefold and 2–threefold, respectively, under dehydration stress (3–12 h). Additionally, *SsRDR1a* and *SsRDR5* expression levels were significantly depressed whereas that of the *SsRDR2b* gene did not significantly change.

### Response of SsAGO, SsDCL and SsRDR expression to *Xanthomonas albilineans* infection

**Figure 8:**
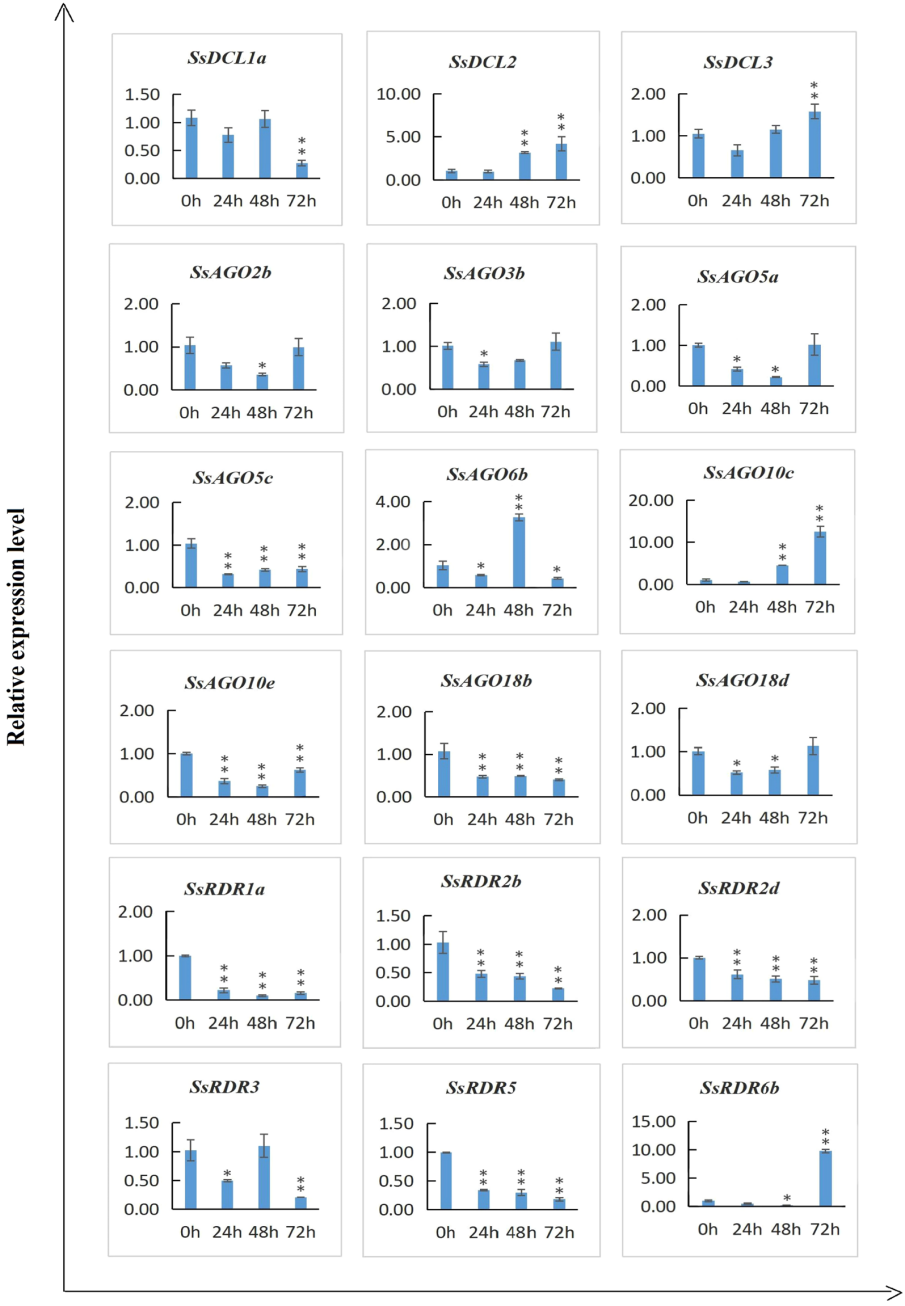
Quantitative real time PCR (qRT-PCR) analysis of relative transcript expression of 18 candidate genes encoding *SsDCL*, *SsAGO*, and *SsRDR* in *S. spontaneum* clone SES208 inoculated with *X. albilineans* strain Xa-FJ1 at 0, 24, 48, and 72 h post-infection (hpi). The x-axis indicates the time points of experimental treatment, while the y-axis indicates the relative expression level. The top young leaves were sampled and used for qRT-PCR assay. Relative transcript expression values are presented as means ± standard errors based on three biological replicates with three technical replicates. Significant differential expression is indicated by an asterisk (*, *p* < 0.05; **, *p* < 0.01).

Expression profiles for the 18 candidate genes were also assessed by qRT-PCR on the young leaves of *S. spontaneum* clone SES208 inoculated with *X. albilineans*. Seven of the *SsAGO* candidate genes had decreased expression levels upon *X. albilineans* infection, with *SsAGO5c*, *SsAGO10e*, and *SsAGO18b* showing significant down-regulation (Fig. 8). *SsAGO10c* expression was highly upregulated with increases of 5–13-fold at 48–72 hpi. Expression of the *SsAGO6b* gene varied, with significant decreases at 24 and 72 hpi but increases at 48 hpi. Of the three *SsDCL* genes tested, expression of *SsDCL1a* was dramatically depressed at 72 hpi, whereas that of *SsDCL2* and *SsDCL3* was highly upregulated with increases of 3–4 folds at 48–72 hpi and ~ twofold at 72 hpi, respectively. Five *SsRDR* genes were downregulated to some extent by *X. albilineans* infection, but *SsRDR6* was significantly depressed at 48 hpi and highly upregulated (tenfold increase) at 72 hpi.

## Discussion

Modern sugarcane cultivars originated from nobilization processes have contributed to worldwide advances in the sugar industry over the last century^25^. Modern sugarcane cultivars have complex genomes that present major challenges for producing a reference sequence^23^. Recently three sugarcane genomes, one *S. spontaneum* clone^28^ and two modern cultivars^27,30^ have been sequenced and assembled to provide valuable reference genomes for identification of gene families and functions in sugarcane. RNA silencing in plants plays an important role in regulating gene expression at different levels via sRNAs^5^. Thus, in this study we performed genome-wide analysis of three gene families involved in RNA silencing, DCL, AGO, and RDR in *S. spontaneum*, and further analyzed the expression profiles of these genes in response to biotic and abiotic stresses.

### Genetic diversity and evolution of DCL, AGO, and RDR gene families in plants

To date, at least 20 plant species have been used to investigate the genetic diversity of the AGO, DCL, and RDR gene families, and in this study we confirmed that members of these families showed obviously species-specific variations (Table S5). The number of genes encoding DCLs varied among plant species from four AtDCLs in *Arabidopsis thaliana*^21^ to eight OsDCLs in rice^44^, millet^47^, and *B. napus*^21^. In *S. spontaneum*, we identified only three classes of DCL genes (*DCL1/2/3*) and found no sequences corresponding to the *DCL4* gene. Similarly, a *DCL4* gene was not identified in *B. oleracea*^21^ or *A. duranensis*^48^. The number of genes encoding AGOs ranged from seven in cucumber^49,50^ to 27 in *B. napus*^21^. Total 21 *SsAGO* genes were identified in *S. spontaneum*. Notably, the monocot plants including rice, maize, and sugarcane have evolved an AGO18 subclade that falls into the AGO1/5/10 clade^51,52^. The minimum number (5) of RDR gene family members was found in seven plants including rice^44^, maize^39^, grapevine^53^, and three legume crops (chickpea, pigeonpea, and groundnut)^48^, whereas *B. napus* had the maximum number (16)^21^. Our findings revealed that DCL4 and AGO1, which are commonly seen in both monocot and dicot plants, were lost in *S. spontaneum*, suggesting that the presence of novel *DCL4*- and *AGO1*-like genes are substitute for the functions of the two genes in *S. spontaneum*.

Large segmental duplications and/or tandem duplications might be responsible for evolution of these three gene families, especially for AGOs in rice, giving rise to redundancy or contrasting functions among *OsAGO* genes^44^. For example, large segmental duplications occurred in *OsDCL2a-2b*, *OsAGO1a-1b*, and *OsAGO13-14*, which localized on different chromosomes in rice. On the other hand, tandem duplications were present in three *OsAGO* gene pairs, *OsAGO4a-15*, *OsAGO2-3*, and *OsAGO11-12*, which localized close to each other on chromosomes 1, 4, and 3, respectively. Notably, large segmental duplication events commonly occurred in the AGOs, DCL, and RDR gene families in *S. spontaneum*, suggesting segmental duplication was an important evolution force for three gene families. However, segmental duplication did not appear in the *OsDCL1* or *OsRDR* genes in rice^44^. Tandem duplication was also another important evolutionary force during evolution of *S. spontaneum AGO* genes, but not for *OsDCLs* or *OsRDRs*. Similarly, no tandem duplication events occurred among *OsDCLs* and *OsRDRs* in rice^44^.

Different RDR-DCL-AGO combinations are synergistically involved in specialized RNA silencing to control invading nucleic acids from endogenous (mainly transposons) or exogenous (mainly viruses) origins, and are mediated by diverse sRNAs such as miRNA, transacting siRNAs (ta-siRNA), natural-antisense-transcript-siRNA (nat-siRNA) and virus-derived siRNAs (vsiRNA)^13,54,55^. For instance, DCL1 and AGO1 mainly participate in the miRNA pathway, but the RDR protein is not necessary for miRNA biogenesis^56^, RDR6, SGS3, AGO1 and DCL4 are the main components of the ta-siRNA pathway at the post-transcriptional level^57^, RDR2, AGO4, and DCL3 are involved in the RNA-directed DNA methylation (RdDM) pathway^58^, DCL2/3/4 and RDR6 are common essential components in antiviral RNA silencing medicated by vsiRNA in *Arabidopsis* and rice, while HSP90-activated AGO1/2/4/5/7/10 is loaded with a vsiRNA in *Arabidopsis* but AGO18 positively regulates AGO1 binding to vsiRNAs by sequestering miR168 in rice^51^. In this study, PPI network analysis also indicated that these core component interactions may participate in various RNA silencing pathways. Notably, the SsAGO18s representing a distinct AGO subfamily specific to monocots also actively interacted with two other components.

### Response of DCL gene family members to drought and bacterial stresses

Four types of Dicer or DCL proteins are key components in miRNA and siRNA biogenesis pathways and mediate conversion of long double-stranded RNAs into mature small RNAs^5,59^. These DCLs (DCL1/2/3/4) play different roles in RNA interference-related processes of small RNA biogenesis in *A. thaliana*: DCL1 produces miRNAs and trigger post-transcriptional gene silencing (PTGS), DCL2 is essential for secondary siRNA-mediated transitive silencing via production of some virus-derived siRNAs; DCL3 produces endogenous RDR2-dependent siRNAs; and DCL4 functions in antiviral defense and development pathways by processing ta-siRNA precursors in the small RNA biogenesis pathway^59–61^. However, DCL2, DCL3, and DCL4 showed functional redundancy in siRNA and tasiRNA production^62,63^.

Here, we found that PEG treatment induced substantial upregulation of *SsDCL1a*, *SsDCL2*, and *SsDCL3*, particularly in *SsDCL2* and *SsDCL3*. These findings are similar to those seen for *ZmDCL2/3b* in maize^39,64^ and *CaDCL1/2/3* in pepper^64^ that showed moderately and significantly, respectively, upregulated expression levels in response to drought conditions. Meanwhile, in the presence of *X. albilineans* infection, *SsDCL2* and *SsDCL3* expression levels were upregulated but *SsDCL1a* was unchanged or even downregulated. Similarly, in *Brassica*, the *BnDCL1a* gene was downregulated to varying degrees at 8 and 16 hpi with *Sclerotinia sclerotiorum*^21^. However, previous studies suggested that DCL1-generated sRNAs can positively regulate antibacterial and antifungal immunity^13^. In addition, a previous study showed that levels of *SlDCL1/2a/2c/2d/3* were significantly upregulated in tomato plants infected with *Tomato yellow leaf curl virus*^65^. In pepper, *CaDCL2/3/4* genes were highly expressed following infection with *Cucumber mosaic virus* (CMV) and particularly with *Potato virus Y* (PVY) or *Tobacco mosaic virus* (TMV)^64^. These findings suggested that DCL2 and DCL3 play positive roles in the response of plants to pathogen infection and abiotic stress, but the role of DCL1 may vary depending on plant species or the nature of biotic and abiotic stresses.

### AGO gene family responses to drought and bacterial stress

AGOs together with small RNAs participate in RNA-induced silencing via cleavage of target mRNAs or blocking their translation^66^. Of the ten AtAGO family members in *Arabidopsis*, AGO2 regulates antibacterial immunity by binding miR393b* to modulate exocytosis of antimicrobial PR proteins via a Golgi-localized SNARE gene MEMB12^67^. AtAGO3 primarily binds 24-nt sRNAs with 5′-terminal adenines^68^. AtAGO5 can bind both viral RNAs and microRNAs to control plant–microbe interactions and plant physiology such as regulation of systemic resistance of *Arabidopsis* against *Potato virus X*^69^. AtAGO6 is involved in siRNA accumulation, RdDM and transcriptional gene silencing^70,71^ whereas AtAGO10 promotes miR165/6 degradation via SDN1 and SDN2 exonucleases^72^. AGO18s is unique to monocots and in rice confers broad-spectrum virus resistance by sequestering host miR168 (microRNA168) or miR528 (microRNA528) following viral infection^73,74^.

Among the nine *SsAGOs* genes identified in this study, transcript levels of five genes (*SsAGO2b/5a/5c/6b/10c*) especially in *SsAGO10c* were highly increased in *S. spontaneum* under dehydration stress. Similar results were reported for genes in other plants subjected to drought stress, such as *SlAGO6* in tomato^65^, *PtAGO5b* gene in poplar^75^, and *CaAGO2* and *CaAGO10b* in pepper^64^. Qin et al. (2018) suggested that *CaAGO10b* might response to osmotic stress of pepper plants by regulating ABA (abscisic acid) responsive genes^64^. On the other hand, expression levels of all the tested *SsAGO* genes, expect for *SsAGO6b/10c*, were significantly depressed to some extent in *S. spontaneum* plants after *X. albilineans* infection. Conversely, among the ten AGOs in *Arabidopsis*, only AGO2 is induced by bacterial infection and AGO2 positively regulates immunity by protein arginine methyltransferase 5 (PRMT5)-mediated dual regulation of this protein as well as associated sRNA levels to ensure appropriate plant immune responses^76^. A study by Qin et al.^64^ revealed that expression of *CaAGO6*, and the *CaAGO10b* gene in particular, was obviously upregulated in pepper upon inoculation with TMV, CMV, or PVY.

Notably, one gene pair, *SsAGO10c/10e*, which had a tandem duplication, exhibited different transcript expression patterns for PEG-treatment and *X. albilineans* infection, indicating that these genes may have evolved by segmental duplication events that were followed by differentiation of expression patterns. Kapoor et al.^44^ also proposed that the expression patterns of most *OsAGO* genes differentiated before their evolution by duplication events (tandem or segmental). For the unique class of AGO18 in monocot plants, the expression of *SsAGO18b* and *SsAGO18d* was significantly depressed in *S. spontaneum* exposed to PEG-treatment stress or *X. albilineans* infection, suggesting these two genes may play a negative role in the response of *S. spontaneum* to biotic or abiotic stresses. However, a previous study showed that AGO18s confers positive regulation in broad-spectrum virus resistance in rice^73,74^. This constricting result may be due to the AGO18s from different plants playing different mechanisms, but additional investigation is needed to determine what roles this protein confers in sugarcane resistance to stress. Very little is known about AGO18 gene functions in monocot plants in response to abiotic stress.

### Response of the RDR gene family to drought and bacterial stresses

RDRs play an important role in vsRNA biogenesis and vsRNA-mediated antiviral defenses in plants^77^. *Arabidopsis* has six AtRDRs, and at least three types act in distinct biological processes such as viral defense and chromatin silencing^44^. Furthermore, binding of transcription factors to the promoters of RDR1-6 genes may play important roles in how various plant species respond to biotic stresses^78^. Our data revealed that expression of three RDR genes, *SsRDR1a/2d/5*, was significantly downregulated, but two (*SsRDR3/6b*) were upregulated and one gene (*SsRDR2b*) was not significantly affected by PEG-treatment stress. In contrast to our results, levels of *SlRDR1* in tomato^65^, *PtRDR1c/1d* in poplar^75^, and *CaRDR1* in pepper^64^ were strongly increased by PEG-treatment stress. Upon *X. albilineans* infection, *SsRDR1a/2b/5* gene expression was significantly downregulated, but the *SsRDR6b* gene was strongly upregulated.

Our findings suggested that SsRDR6 plays an important role in defenses against biotic and abiotic stresses in *S. spontaneum*. Similar results were seen for pepper, in which *CaRDR6* gene expression levels were highly increased in the presence of three biotic stresses (CMV, PVY, and TMV inoculation)^64^. In contrast, *Arabidopsis* RDR6 acts as a novel negative regulator of pattern-triggered immunity (PTI) and an *rdr6* mutant exhibits enhanced basal resistance towards a virulent *Pseudomonas syringae* strain^79^. Previous observations showed that RDR6 in *Nicotiana attenuata* played an important role when plants respond to challenges in their native environments^80^. In rice, *OsRDR6* is responsible for the observed ABA (abscisic acid)-mediated amplification and silencing of RDR6-dependent siRNA transcripts^81^.

## Conclusions

In this study we identified 21 SsAGOs, four SsDCLs and 11 SsRDRs in the *S. spontaneum* genome. Genes in these three families present characteristic conserved domains and *cis-*elements as well as distinct expression profiles. Chromosome localization analysis revealed that segmental and/or tandem duplication contributed to the evolution of these genes, particularly for the SsAGO family. RNA-seq data analysis indicated tissue-specific expression patterns for some genes such as *SsRDR5*, which showed preferential expression in leaves, whereas *SsAGO18c*, *SsAGO18b*, *SsAGO10e*, and *SsAGO6b* exhibited stem-specific expression. Additionally, qRT-PCR analysis indicated that the expression patterns of some genes in three families differed in *S. spontaneum* plants exposed to PEG-treatment stress or *X. albilineans* infection in that *SsAGO10c*, *SsDCL2*, and *SsRDR6b* genes were strongly upregulated and *SsAGO18b*, *SsRDR1a*, *SsRDR2b/2d*, and *SsRDR5* were significantly depressed under the two stresses. Our findings can enhance our knowledge of the roles of these genes encoding SsAGOs, SsDCLs, and SsRDRs in biotic and abiotic stress response of sugarcane plants.

## Supplementary information

Supplementary Information 1.

Supplementary Information 2.

Supplementary Information 3.

Supplementary Information 4.

## Data Availability

All data, including image files, are available from the corresponding author on reasonable request.
