# Supplementary material for: Genome-wide identification and characterization of DCL, AGO and RDR gene families in Saccharum spontaneum
Source: Sci Rep. 2020 Aug 6;10:13202. doi: 10.1038/s41598-020-70061-7 (PMC7413343; doi:10.1038/s41598-020-70061-7)
Supplement: Supplementary file 1 — Supplementary Information 1. [file 41598_2020_70061_MOESM1_ESM.pdf]

Genome-wide identification and characterization of DCL, AGO and RDR gene families in *Saccharum spontaneum*

Dong-Li Cui[1], Jian-Yu Meng[1], Xiao-Yan Ren[2], Jing-Jing Yue[3], Hua-Ying Fu[1], Mei-Ting Huang[1], Qing-Qi Zhang[2] and San-Ji Gao[1, *]

[1] National Engineering Research Center for Sugarcane, Fujian Agriculture and Forestry University, Fuzhou, Fujian 350002, China

[2] College of Agriculture, Fujian Agriculture and Forestry University, Fuzhou, Fujian 350002, China

[3] FAFU and UIUC-SIB Joint Center for Genomics and Biotechnology, Fujian Provincial Key Laboratory of Haixia Applied Plant Systems Biology, Fujian Agriculture and Forestry University, Fuzhou, Fujian 350002, China

* Correspondence should be addressed to Qing-Qi Zhang, 000q010002@fafu.edu.cn; San-Ji Gao, gaosanji@fafu.edu.cn

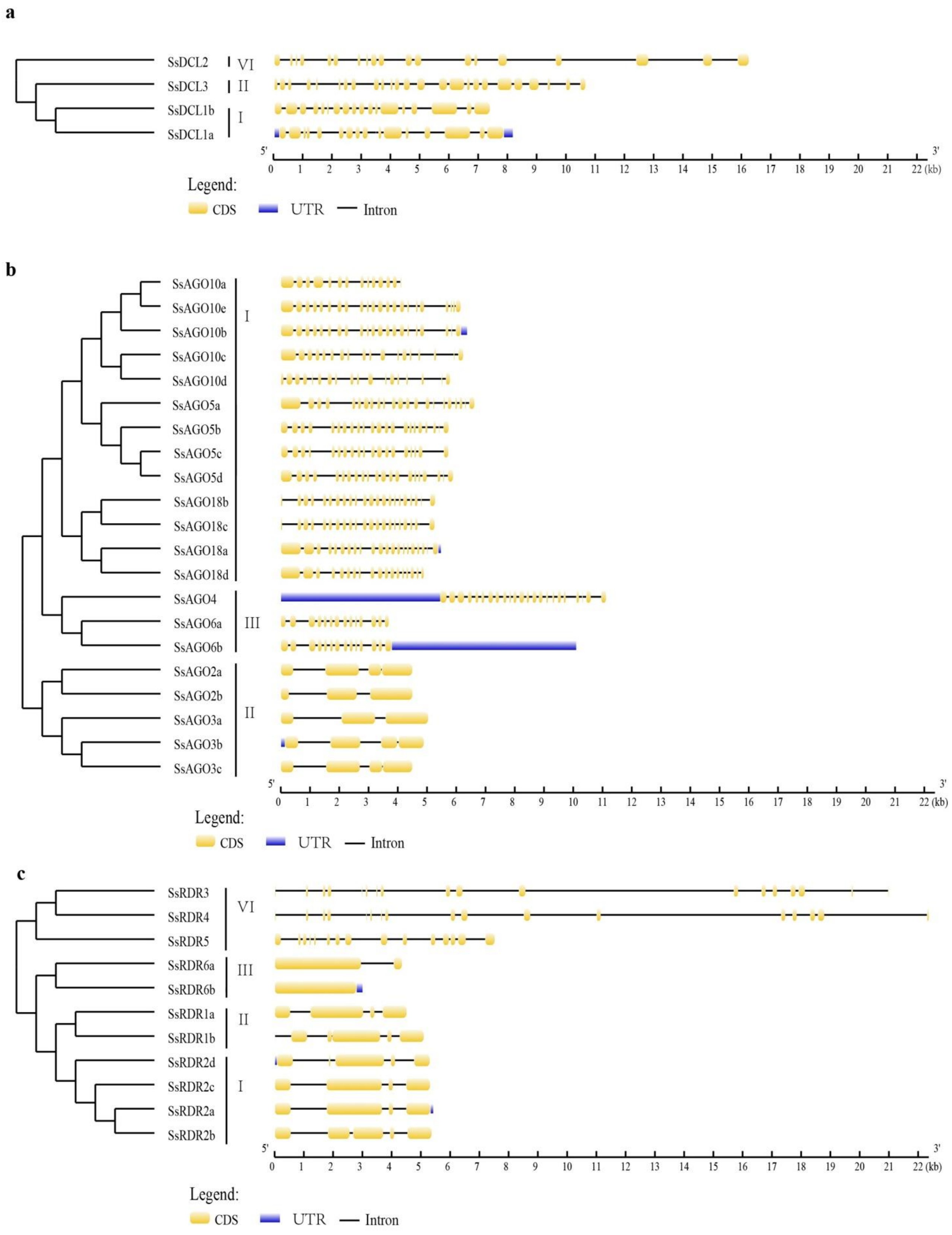

Supplementary Figure S1. Structure analysis of genes encoding DCL (a), AGO (b), and RDR (c) families in *S. spontaneum*. The full-length cDNA sequences of each gene are analyzed and displayed. Coding sequences (CDS) and untranslated regions (UTR) are indicated by yellow and blue boxes, respectively. Introns are indicated by black lines. The scale bar at the bottom indicates the length of the genes in kilobase (kb).

(a) Dicer-like (DCL)

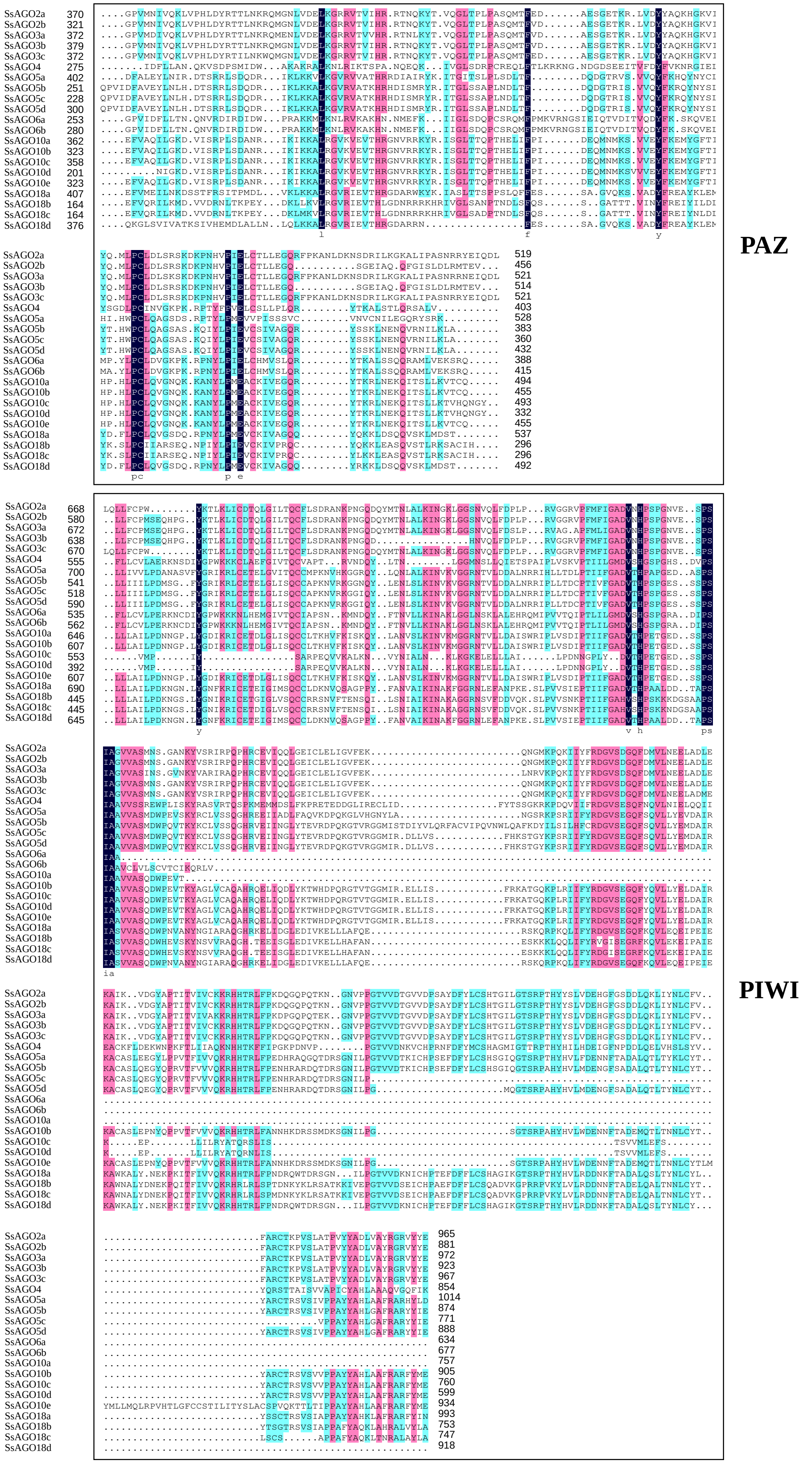

(b) Argonaute (AGO)

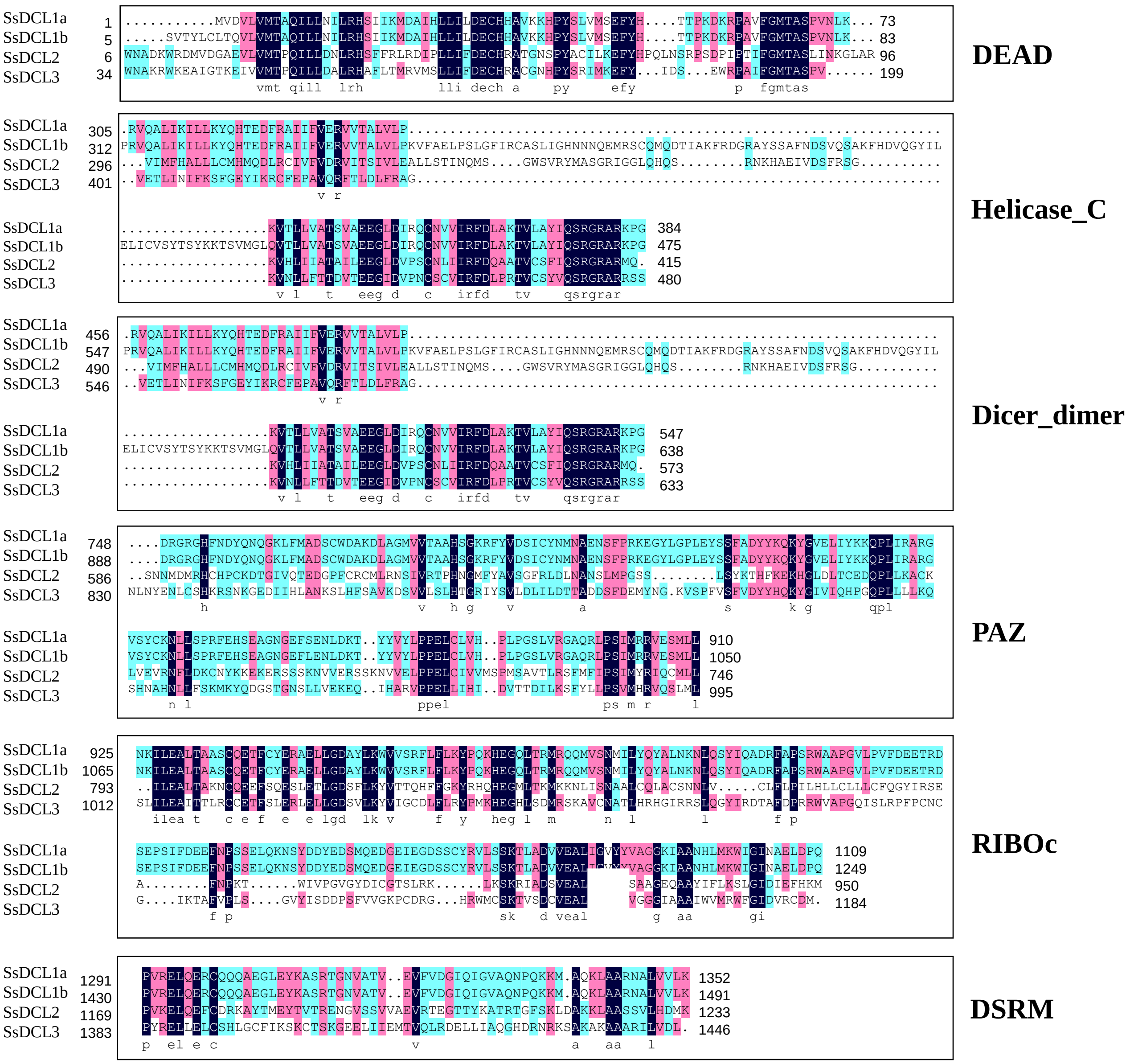

(c) RNA dependent RNA Polymerase (RDR)

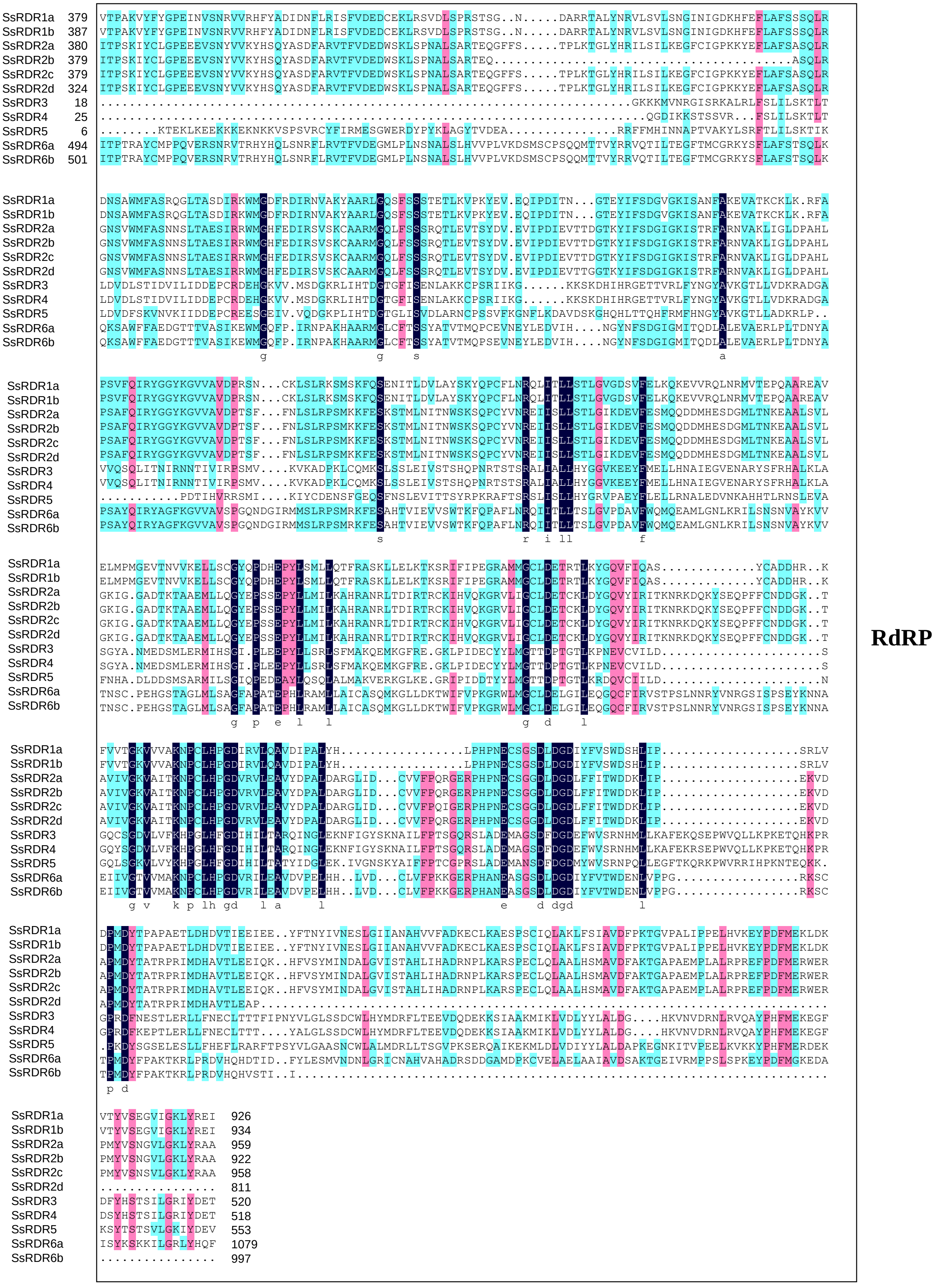

Supplementary Figure S2. Pairwise alignment of amino acid sequence of conserved domains in DCL (a), AGO (b), and RDR (c) families of *S. spontaneum.*

Supplementary Table S1. Primers used for 18 candidate genes encoding DCL, AGO, and RDR families in *S. spontaneum*.

Gene name [a]	Gene ID	Forward primer (5'→3')	Reverse primer (5'→3')
SsDCL3	Sspon.01G0019860-4D	TAAAGGTGGACCTCGGACTG	CAGCAGGTATGTGCAAGGTG
SsDCL2	Sspon.01G0022370-1A	ATGAAACGGCTACTGGAACC	CCGAGTGGCCTTGTATGTAG
SsDCL1a	Sspon.01G0001230-2C	GTCACTGAGGAGAAGGCAAG	GCACCAGTTTCAAGGAAAGC
SsAGO5c	Sspon.01G0014460-1A	CCATCAGAGAGGGAGGGTAG	ATTGTCCGATGGAAGGGTTG
SsAGO5a	Sspon.01G0028360-2B	ACTCTGGCATTCAGGGAACG	AGACTGATCGTGTGCATCGG
SsAGO10e	Sspon.08G0006580-3C	ACTCACCGGACATCAGGAAG	CGACAATCCCATCTGTGTCG
SsAGO18b	Sspon.01G0024300-1A	CACTGCACGTCCAGATCAAG	CAACACTGTGACACGAGACC
SsAGO18d	Sspon.02G0007830-2D	ACGTGACCATCTCACCAGAG	TCTGCCTGTTCTCGGATACC
SsAGO3b	Sspon.05G0030950-1C	TGATCCAGCAGCTTGGTGAG	GATCTTCTGCGGCTTCATGC
SsAGO2b	Sspon.05G0037340-1D	AGCAGTTTACGGCGTCTTTC	AAGACCCTGCAAGACCTCTC
SsAGO6b	Sspon.07G0020900-2D	CTGCTGGAGCTGTCCAAGTG	GGCTTTCCTTCACGACCGTT
SsAGO10c	Sspon.08G0006580-2B	CCACCTCAGGCAGTTCATCG	GCTTCCTGATGTCCGGTGAG
SsRDR1a	Sspon.04G0003980-2D	CCATCTGTGCTGTCAAGCTC	GGAATAATGTCCCGCTCTGC
SsRDR2b	Sspon.05G0009620-2B	AAGAAGCCGCTTTGTCTGTC	TCAGCCTATTAGCCCGATGG
SsRDR6b	Sspon.08G0019290-2D	GGAACAACAACTGTCGCAAG	TCACTAGGCTGCATTGTCAC
SsRDR2d	Sspon.05G0009620-4D	GGAAAGGCCTCATCCAAATG	TCAACCTTCTCCGGAATCAG
SsRDR5	Sspon.03G0023160-2C	GGCGTATTTGCAATCTCAGC	GAAAGTTGGCCGTTGTCAAG
SsRDR3	Sspon.05G0014430-3C	ACAGGACATCAACGTCAAGG	CCAGATGCAAGCTTTAGTGC
GAPDH		CACGGCCACTGGAAGCA	TCCTCAGGGTTCCTGATGCC

[a] GAPDH (reference gene), glyceraldehyde 3-phoshate dehydrogenase.

Supplementary Table S5. The number of genes encoding DCL, AGO, and RDR proteins identified in 21 plant species.

Plant species	DCL	AGO	RDR	Reference
Arabidopsis	4	10	6	Vazquez 2006
rice	8	19	5	Kapoor et al. 2008
maize	5	18	5	Qian et al. 2011
tomato	7	15	6	Bai et al. 2012
grapevine	4	13	5	Zhao et al. 2015
soybean	7	21	7	Liu et al. 2014
sorghum	5	14	7	Liu et al. 2014
foxtail millet	8	19	11	Yadav et al. 2015
poplar	5	15	9	Zhao et al. 2015
B. napus	8	27	16	Cao et al. 2016
B. rapa	4	13	6	Cao et al. 2016
B. oleracea	4	14	7	Cao et al. 2016
cucumber	5	7	8	Gan et al. 2016 & 2017
chickpea	4	13	5	Garg et al. 2017
pigeonpea	4	13	5	Garg et al. 2017
A. duranensis	3	11	5	Garg et al. 2017
A. ipaensis	4	11	5	Garg et al. 2017
coffee	9	11	8	Noronha et al. 2017
pepper	4	12	6	Qin et al. 2018
Citrus sinensis	5	13	7	Sabbione et al. 2019
S. spontaneum	4	21	11	In this study

References

1. Bai, M.; Yang, G.S.; Chen, W.T.; Mao, Z.C.; Kang, H.X.; Chen, G.H.; Yang, Y.H.; Xie, B.Y. Genome-wide identification of Dicer-like, Argonaute and RNA-dependent RNA polymerase gene families and their expression analyses in response to viral infection and abiotic stresses in Solanum lycopersicum. *Gene* **2012**, *501*, 52–62.
2. Cao, J.Y.; Xu, Y.P.; Li, W.; Li, S.S.; Rahman, H.; Cai, X.Z. Genome-wide identification of Dicer-Like, Argonaute, and RNA-Dependent RNA Polymerase gene families in *Brassica* species and functional analyses of their *Arabidopsis* homologs in resistance to *Sclerotinia sclerotiorum*. *Front. Plant Sci.* **2016**, *7*, 1614.
3. Gan D. F.; Liang D. D.; Wu J.; Zhan M. D.; Yang F.; Xu W. J. Genome-wide identification of the Dicer-Like, Argonaute, and RNA-dependent RNA polymerase gene families in cucumber (*Cucumis sativus* L.). *J. Plant Growth Regul.* **2016**, 35, 135–150.
4. Gan, D.; Zhan, M.; Yang, F.; Zhang, Q.; Hu, K.; Xu, W.; Lu, Q.; Zhang, L.; Liang, D. Expression analysis of argonaute, Dicer-like, and RNA-dependent RNA polymerase genes in cucumber (*Cucumis sativus* L.) in response to abiotic stress. *J. Genet.* **2017**, *96*, 235–249.

5. Garg, V.; Agarwal, G.; Pazhamala, L.T.; Nayak, S.N.; Kudapa, H.; Khan, A.W.; Doddamani, D.; Sharma, M.; Kavi, K.P.; Varshney, R.K. Genome-wide identification, characterization, and expression analysis of small RNA biogenesis purveyors reveal their role in regulation of biotic stress responses in three legume crops. *Front. Plant Sci.* **2017**, *8*, 488.
6. Kapoor, M.; Arora, R.; Lama, T.; Nijhawan, A.; Khurana, J.P.; Tyagi, A.K.; Kapoor, S. Genome-wide identification, organization and phylogenetic analysis of Dicer-like, Argonaute and RNA-dependent RNA Polymerase gene families and their expression analysis during reproductive development and stress in rice. *BMC Genomics* **2008**, *9*, 451.
7. Liu, X.; Lu, T.; Dou, Y.; Yu, B.; Zhang, C. Identification of RNA silencing components in soybean and sorghum. *BMC Bioinformatics* **2014**, *15*, 4.
8. Noronha, F.C.; Marinho, R.P.; Cherubino, R.T.; Ricon, D.O.R.; Cunha, D.S.C.T.; Rodrigues, D.A.L.; de Souza, G.M.; Chalfun-Junior, A. A genome-wide analysis of the RNA-guided silencing pathway in coffee reveals insights into its regulatory mechanisms. *PLOS ONE* **2017**, *12*, e176333.
9. Qian, Y.; Cheng, Y.; Cheng, X.; Jiang, H.; Zhu, S.; Cheng, B. Identification and characterization of Dicer-like, Argonaute and RNA-dependent RNA polymerase gene families in maize. *Plant Cell Rep.* **2011**, *30*, 1347–1363.
10. Qin, L.; Mo, N.; Muhammad, T.; Liang, Y. Genome-wide analysis of DCL, AGO, and RDR gene families in Pepper (*Capsicum Annuum L.*). *Int. J. Mol. Sci.* **2018**, *19*, 1038.
11. Sabbione, A.; Daurelio, L.; Vegetti, A.; Talon, M.; Tadeo, F.; Dotto, M. Genome-wide analysis of AGO, DCL and RDR gene families reveals RNA-directed DNA methylation is involved in fruit abscission in *Citrus sinensis*. *BMC Plant Biol.* **2019**, *19*, 401.
12. Vazquez, F. *Arabidopsis* endogenous small RNAs: highways and byways. *Trends Plant Sci.* **2006**, *11*, 460–468.
13. Yadav, C.B.; Muthamilarasan, M.; Pandey, G.; Prasad, M. Identification, characterization and expression profiling of Dicer-Like, Argonaute and RNA-Dependent RNA Polymerase gene families in Foxtail Millet. *Plant Mol. Biol. Report* **2015**, *33*, 43–55.
14. Zhao, H.; Zhao, K.; Wang, J.; Chen, X.; Chen, Z.; Cai, R.; Xiang, Y. Comprehensive analysis of Dicer-Like, Argonaute, and RNA-dependent RNA Polymerase gene families in Grapevine (*Vitis Vinifera*). *J. Plant Growth Regul.* **2014**, *34,* 108–21.
15. Zhao, K.; Zhao, H.; Chen, Z.; Feng, L.; Ren, J.; Cai, R.; Xiang, Y. The Dicer-like, Argonaute and RNA-dependent RNA polymerase gene families in *Populus trichocarpa*: gene structure, gene expression, phylogenetic analysis and evolution. *J. Genet.* **2015**, *94*, 317–321.
